# The spatio-temporal relationship between white matter lesion volume changes and brain atrophy in clinically isolated syndrome and early multiple sclerosis

**DOI:** 10.1016/j.nicl.2022.103220

**Published:** 2022-10-03

**Authors:** Rozemarijn M. Mattiesing, Giordano Gentile, Iman Brouwer, Ronald A. van Schijndel, Bernard M.J. Uitdehaag, Jos W.R. Twisk, Ludwig Kappos, Mark S. Freedman, Giancarlo Comi, Dominic Jack, Nicola De Stefano, Frederik Barkhof, Marco Battaglini, Hugo Vrenken

**Affiliations:** a MS Center Amsterdam, Radiology and Nuclear Medicine, Amsterdam Neuroscience, Amsterdam UMC Location VUmc, De Boelelaan 1118, 1081 HZ Amsterdam, the Netherlands; b Department of Medicine, Surgery and Neuroscience, University of Siena, 53100 Siena, Italy; c Siena Imaging SRL, 53100 Siena, Italy; d MS Center Amsterdam, Neurology, Amsterdam Neuroscience, Amsterdam UMC Location VUmc, De Boelelaan 1118, 1081 HZ Amsterdam, the Netherlands; e Epidemiology and Data Science, Amsterdam UMC Location VUmc, De Boelelaan 1118, 1081 HZ Amsterdam, the Netherlands; f Neurologic Clinic and Policlinic, Departments of Clinical Research and Biomedical Engineering, University Hospital Basel, 4031 Basel and University of Basel, 4001 Basel, Switzerland; g University of Ottawa, Department of Medicine, Ottawa ON, K1N 6N5 and the Ottawa Hospital Research Institute, Ottawa, ON K1H 8L6, Canada; h Università Vita Salute San Raffaele, Casa di Cura del Policlinico, 20132 Milan, Italy; i Merck Serono Ltd, Feltham, TW14 8HD, UK, an affiliate of Merck KGaA; j UCL Institutes of Neurology and Healthcare Engineering, London, WC1E 6BT, UK

**Keywords:** CDMS, clinically definite multiple sclerosis, CIS, clinically isolated syndrome, DT, delayed treatment, ET, early treatment, GM, gray matter, LCM, lesion change map, MS, multiple sclerosis, PBVC, percentage brain volume change, PD, proton-density, PVVC, percentage ventricular volume change, sc IFN β-1a, subcutaneous interferon beta-1a, SubIs, subtraction images, TLVC, total lesion volume change, WM, white matter, Brain atrophy, White matter lesions, Interferon beta-1a, Magnetic resonance imaging, Early multiple sclerosis

## Abstract

**Background:**

White matter lesions and brain atrophy are both present early in multiple sclerosis. However, the spatio-temporal relationship between atrophy and lesion processes remains unclear.

**Methods:**

Yearly magnetic resonance images were analyzed in 392 patients with clinically isolated syndrome from the 5-year REFLEX/REFLEXION studies. Patients received early treatment (from baseline; N = 262) or delayed treatment (from month-24; N = 130) with subcutaneous interferon beta-1a. Global and central atrophy were assessed using FSL-SIENA to provide yearly percentage volume change of brain and ventricles, respectively. Yearly total lesion volume change was calculated by subtracting the sum of the negative lesion volume change (disappearing + shrinking) from the positive lesion volume change (new + enlarging) for each yearly interval, as determined by an in-house developed semi-automated method. Using linear mixed models, during the period where patients had received ≥1 year of treatment, we investigated whether total lesion volume change was associated with percentage brain volume change or percentage ventricular volume change in the next year, and vice versa.

**Results:**

Higher total lesion volume change was related to significantly faster global atrophy (percentage brain volume change) in the next year (B = − 0.113, SE = 0.022, p < 0.001). In patients receiving early treatment only, total lesion volume change was also associated with percentage ventricular volume change in the next year (B = 1.348, SE = 0.181, p < 0.001). Voxel-wise analyses showed that in patients receiving early treatment, higher total lesion volume change in years 2, 3, and 4 was related to faster atrophy in the next year, and in year 4 this relationship was stronger in patients receiving delayed treatment. Interestingly, faster atrophy was related to higher total lesion volume change in the next year (percentage brain volume change: B = − 0.136, SE = 0.062, p = 0.028; percentage ventricular volume change: B = 0.028, SE = 0.008, p < 0.001).

**Conclusions:**

Higher lesion volume changes were associated with faster atrophy in the next year. Interestingly, there was also an association between faster atrophy and higher lesion volume changes in the next year.

## Highlights

• Relations between atrophy and lesions in early multiple sclerosis were investigated.
• Higher white matter lesion volume changes were related to faster subsequent atrophy.
• Future studies need to determine if these processes are related or just simultaneous.

## 1 Introduction

Multiple sclerosis (MS) is a chronic demyelinating inflammatory disease of the central nervous system with a neurodegenerative component. The accumulation of white matter (WM) lesions and accelerated regional and global atrophy, both of which are present in the brain from the early stages of the disease, are among the most relevant pathological processes (Simon, 2014). The development of these two pathological processes are presumed to be interrelated (Fisher et al., 2002) but the underlying mechanisms remain to be elucidated.

To effectively intervene and target the underlying pathologies in the early phase of the disease, it is crucial to broaden our understanding of the underlying mechanisms, their interactions, and whether these can be modified by early treatment. For this reason, it is especially important to uncover the association between inflammation and neurodegeneration in patients with clinically isolated syndrome (CIS) and early MS.

There are relatively few longitudinal studies of the relationship between atrophy and WM damage in patients with CIS and early MS (Chard et al., 2003, Dalton et al., 2002, Dalton et al., 2004, Paolillo et al., 2004, Varosanec et al., 2015), and these studies have focused on the assumption that inflammation precedes neurodegeneration. For example, Chard et al. (2003) found that, in long-term follow-up of patients with CIS, subsequent atrophy was more strongly related to the accumulation of focal T2 lesions in the early phase (0–5 years) rather than later phases in the study (5–10 years and 10–14 years). Dalton et al. (2002) found that baseline lesion measures are related to the development of subsequent ventricular enlargement over one year in those with CIS. According to a review by Zivadinov and Zorzon (2002), inflammatory damage and ongoing WM changes, such as gadolinium enhancing lesions, seem to be predictive of later atrophy in relapsing remitting MS.

Currently it is unknown whether neurodegeneration in MS is secondary to the inflammatory processes leading to WM lesions or whether neurodegeneration is a primary disease process, that (also) leads to secondary focal or diffuse WM damage. Alternatively, both options might occur simultaneously (for gray matter atrophy this has been described [Calabrese et al. (2015)]). A recent review concluded that evidence from animal models and genetic studies favors a pathogenesis in which inflammation precedes neurodegeneration (Milo et al., 2020). However, few patient studies have investigated the possibility that atrophy might precede WM damage.

To investigate how WM lesions and atrophy in MS develop over time and how their evolution is related, long-term follow-up with regular MRI is required. The randomized, double-blind, placebo-controlled, multicenter REFLEXION clinical study provides such an opportunity. In this study, patients presenting with CIS were followed over a period of 5 years with yearly MRI scans. In the primary analyses of the study by Comi et al. (2017), overall MRI activity was reduced in patients receiving early treatment compared to patients receiving delayed treatment with subcutaneous interferon beta-1a (sc IFN β-1a). However, that study did not look at the relationship between atrophy and WM lesion measures.

In the current study, we therefore conducted advanced post-hoc image analyses on the REFLEXION dataset. Our main objective was to investigate whether WM lesion changes are spatio-temporally related to subsequent atrophy and, conversely, whether atrophy is related to the development of subsequent WM lesion changes in patients with CIS and early MS. In turn, we also studied if these possible associations differed between patients receiving either early or delayed treatment with sc IFN β-1a, or between patients who converted to clinically definite MS during the study and those who did not.

## 2 Methods

### 2.1 Study

REFLEXION (NCT00813709) was a preplanned extension of the randomized, double-blind, placebo-controlled, multicenter REFLEX clinical study (REbif FLEXible Dosing in Early MS; NCT00404352). Procedures and the design of the study have been described in detail previously (Comi et al., 2012, Comi et al., 2017). Briefly, patients with CIS at high risk of converting to MS were included and either randomized to one of two early treatment arms, where treatment with sc IFN β-1a 44 μg was initiated once a week or three times a week, or to the delayed treatment arm in which, during the first 2 years (i.e., the REFLEX phase), patients did not receive treatment (placebo group) but at the start of REFLEXION received sc IFN β-1a 44 μg three times a week. If patients converted to clinically definite MS (CDMS), they received open-label treatment with sc IFN β-1a 44 μg three times a week. CDMS was defined by a relapse accompanied by an abnormal MRI scan or a sustained increase in Expanded Disability Status Scale score of ≥1.5 points. For the purpose of this study, the sc IFN β-1a 44 μg once a week and three times a week treatment arms were considered together.

### 2.2 MRI data

For the current post-hoc analyses, multicenter yearly MRI scans over the full REFLEX/REFLEXION study period of 5 years were evaluated. These consisted of 1 × 1 × 3 mm^3^ 2D dual-echo proton-density (PD)-/T2-, and T1-weighted images. The Image Analysis Center of Amsterdam UMC (Location VUmc, Amsterdam, the Netherlands) provided manual delineations of the PD-/T2-weighted lesions for each yearly visit and manually edited T1-weighted brain extraction masks originally obtained by using the FSL brain extraction tool (Smith, 2002), part of FMRIB’s software library (Smith et al., 2004). Scans were included if the input data were of sufficient quality and the different processing steps and output of the image analyses described below passed quality control measures (specific criteria are described in more detail in Section 3. Results).

### 2.3 Longitudinal atrophy measurement

The T1-weighted images were corrected for slice-to-slice intensity variations (due to interleaved acquisitions) and subsequently lesion filled with the linearly registered PD-/T2-weighted manual delineations. Yearly percentage brain volume change (PBVC) and percentage ventricular volume change (PVVC) were estimated with SIENA (Smith et al., 2001, Smith et al., 2002) and its extension VIENA (Vrenken et al., 2014), both part of FMRIB’s software library (Smith et al., 2004). The normalized and lesion filled T1-weighted image and the manually edited brain mask were used as input. Yearly PBVC and PVVC were used as longitudinal measures of global atrophy and central atrophy, respectively.

### 2.4 Lesion volume change quantification

Yearly lesion volume changes were quantified by an in-house developed semi-automated method that is based on the use of subtraction images (SubIs) (Moraal et al., 2010a, Moraal et al., 2010b). The details of this method are described in Battaglini et al. (2022). Briefly, before creating the SubIs, the slice-to-slice variation in signal intensity on the PD-weighted images was corrected. Then the PD images of both visits of a yearly interval (e.g., baseline-month 12) were registered to a common halfway space using a similar procedure to that used in SIENA software, based on the T2-weighted images. To obtain the SubIs, the PD-weighted image of the first visit of each interval was subtracted from the second visit. To give a robust analysis, the SubIs were further normalized to account for the differences between the study sites and MRI scanners from which the images were obtained. Voxels inside the lesion masks with a normalized intensity difference exceeding 1.5 standard deviations (|Z| > 1.5) were labeled as changing. Based on the baseline and follow-up lesion masks and these voxel-wise lesion changes, each individual lesion was labeled as new, enlarging, shrinking, or disappearing. The yearly total lesion volume change (TLVC) was thereafter calculated by subtracting the sum of the negative lesion volume change (disappearing + shrinking) from the positive lesion volume change (new + enlarging) for each interval. To allow lesion probability map analyses of the anatomical distribution of the four lesion types, lesion labels were stored in a lesion change map (LCM) for each of the four lesion types, for each patient and for each interval.

### 2.5 Voxel-wise input images

The following steps were performed to produce the input images for voxel-wise statistical analyses. First, we created a study-specific template (see Supplementary Fig. 1). FSL-SIENAX (Smith et al., 2002) was used to obtain normalized brain volume for all baseline T1-weighted images. Afterwards, 100 patients were selected based on the percentile distribution of normalized brain volume (from 1st to the 100th percentile). For each of these 100 patients, the T1-weighted images were intensity-normalized (divided by the 99th intensity percentile of the non-zero voxels and multiplied by 10,000), non-linearly registered to the MNI standard space (resolution = 2 × 2 × 2 mm^3^) using FSL-FNIRT (Andersson et al., 2007) and averaged to create the study-specific template. Second, to ensure that all the images of each patient underwent the same preprocessing and to avoid interpolation bias, a patient-specific template was created (see Supplementary Fig. 2). Accordingly, the T1-weighted images were intensity-normalized using the N4 algorithm (Tustison et al., 2010), linearly registered to the baseline T1-weighted scan using FLIRT (Jenkinson et al., 2002), and averaged to create the patient-specific template. The patient-specific template was then non-linearly registered on the study-specific template space using FNIRT and the warp-fields generated from this registration were used for the subsequent registration of SIENA and LCM outputs on the study-specific template.

To study local atrophy, brain edge shift maps were created. For each patient, the yearly brain edge shift images provided by SIENA were spatially dilated, non-linearly registered to the study-specific template using FNIRT, masked with a standard space brain edge image, smoothed with an isotropic Gaussian kernel with a sigma of 5 mm, and remasked (Bartsch et al., 2007, De Stefano et al., 2003) (see Supplementary Fig. 3).

To study local lesion activity, the yearly LCMs of enlarging, new, shrinking, and disappearing lesions were non-linearly registered to the study-specific template using the warp-fields generated through the procedure described above (see Supplementary Fig. 4).

### 2.6 Statistical analyses

#### 2.6.1 Whole brain statistical analyses

Statistical analyses were performed in RStudio. For the whole brain analyses, linear mixed models were used to deal with the repeated measurements we have for the yearly atrophy and lesion change measures. We incorporated a three-level structure where observations were clustered within the patients and the patients were clustered within the different study sites. All linear mixed models were corrected for age and sex. Regarding conversion to CDMS, depending on the research question, we categorized patients into converters and non-converters either considering the full 5-year study period or using each patient’s time-dependent CDMS status for the yearly interval under consideration. An alpha of 0.05 was used as the cut-off for significance.

First, we performed separate analyses to assess if atrophy (PBVC and PVVC) and lesion volume changes (TLVC) differed between treatment groups and between converters and non-converters. Linear mixed models were used with treatment and interval-specific CDMS status as fixed factors (full model details are listed in Supplementary Table 1: models 1–3). To assess the treatment effect at the different time-points, we used a similar model but incorporated an interaction between treatment and the yearly intervals (Supplementary Table 1: models 4–6). For atrophy, PBVC and PVVC were alternately used as the dependent variable; for lesion volume changes, TLVC was the dependent variable. Second, to analyze whether lesion volume changes were related to atrophy in the next year, we incorporated a time-lag in our linear mixed models to link TLVC in year 1 to PBVC or PVVC in year 2, and TLVC in year 2 to PBVC or PVVC in year 3, etc. (see Supplementary Table 2). We did this by applying a linear mixed model with TLVC as the independent variable and PBVC or PVVC in the next year as the dependent variable (Supplementary Table 1: models 7 and 8). In the time-lag models we adjusted for treatment and CDMS status across the whole study period. Additionally, to analyze whether the relationship between lesion volume changes and atrophy in the next year differed between treatment groups or between converters and non-converters, we used similar models but also incorporated an interaction between treatment and TLVC (Supplementary Table 1: models 9 and 10), and CDMS status across the whole study period and TLVC (Supplementary Table 1: models 11 and 12), respectively.

To prevent confounding by resolving edema and pseudo-atrophy at the start of treatment, all linear mixed models were performed on the data points where the patients have received at least one year of treatment. This means that for the early treatment group, TLVC in years 2, 3, and 4 and PBVC or PVVC in the next year were included; and for the delayed treatment group, only TLVC in year 4 and PBVC or PVVC in year 5 (Supplementary Table 2). This will be called the stable treatment period.

To analyze the relationship between lesion volume changes and atrophy in the next year in an untreated period, we included the data points of TLVC in year 1 and PBVC or PVVC in year 2 of the delayed treatment (then placebo) group in the REFLEX period (Supplementary Table 2), while excluding the delayed treatment patients who converted during the first 2 years of the study and controlling for CDMS status across the whole study period (Supplementary Table 1: models 13 and 14). To analyze whether the relationship differed between the REFLEX placebo period and the REFLEXION treatment period (TLVC in year 4 and PBVC or PVVC in year 5; Supplementary Table 2) excluding the first year of treatment of the delayed treatment patients (TLVC in year 3 and PBVC or PVVC in year 4), we incorporated an interaction between TLVC and period and corrected for CDMS status across the study period in a separate linear mixed model (Supplementary Table 1: models 15 and 16).

Third, to analyze whether changes in atrophy were related to lesion volume changes in the next year under stable treatment and in the placebo period, we repeated the time-lag analyses described above but used PBVC or PVVC as independent variable and TLVC in the next year as the dependent variable (Supplementary Table 1: models 17–26).

#### 2.6.2 Voxel-wise statistical analyses

For each yearly interval, regional statistical inference was carried out using permutation testing (5000 permutations) (Nichols and Holmes, 2002) as implemented in the *Randomise* program of FMRIB’s software library. The Threshold-Free Cluster Enhancement randomise option was used. When looking at the difference between early and delayed treatment patients and converters/non-converters in terms of atrophy and LCMs within each interval, design matrixes within the general linear model framework were used with treatment and interval-specific CDMS status as variables of interest, and age, sex, and study site as covariates. TLVC was used as regressor when looking at the relationship with brain edge shifts in the next year. To avoid reporting results that are not biologically meaningful, we selected the 25th percentile of the distribution of the sizes of the significant clusters as a threshold. Therefore, only results with at least 15 significant voxels (p < 0.05) were reported. The anatomical location was determined by using pre-defined standard space masks (see https://www.fmrib.ox.ac.uk/fsl), as provided by the MNI structural atlas and the JHU WM tractography atlas. The number (V) and the location of significant voxels were reported. The voxel-wise analyses were matched to the whole brain analyses but could only be performed for each yearly interval.

#### 2.6.3 Ethics approval

This post-hoc study used data from the REFLEX and REFLEXION studies, which were undertaken in compliance with the Declaration of Helsinki and standards of Good Clinical Practice according to the International Conference on Harmonization of Technical Requirements for Registration of Pharmaceuticals for Human Use. Before initiation of the studies at each center, the relevant institutional review board or independent ethics committee reviewed and approved the study protocols, patient information leaflets, informed consent forms, and investigator brochures. All patients provided written informed consent at the screening visit of REFLEX, and before enrollment to REFLEXION.

## 3 Results

A total of 400 patients enrolled in the REFLEXION study provided MRI data for the extension period, and the input data of 392 patients were included in the current analyses (Table 1). Concerning the input data, four patients were excluded because of incomplete study data, two patients because of an inconsistent acquisition protocol, and two patients because no consecutive visits were available that were needed to calculate yearly atrophy and lesion change measures. Regarding visit data, five visits were excluded because of incorrect/incomplete image(s), eight visits because of movement, six visits because of missing data, and two visits were excluded from the lesion change analyses because of corrupted PD-weighted images. The quality check of the output from the lesion change quantification and longitudinal atrophy measures resulted in 158 excluded MRI measurements, corresponding to 23 lesion change quantification, 133 longitudinal atrophy, and 2 shared rejections. Reasons for exclusion were: low quality of the images, artifacts, registration problems, and pipeline failure.

**Table 1:** Demographics of the included patients.

	Converters to CDMS	Non-converters to CDMS	Early treatment	Delayed treatment	Overall
Patients (N)	162	230	262	130	392
Gender (F/M)	95/67	147/83	162/100	80/50	242/150
Age, y (mean ± SD)	30.32 ± 7.99	32.23 ± 8.52	31.68 ± 8.43	30.97 ± 8.19	31.44 ± 8.35

CDMS = clinically definite multiple sclerosis (across the whole study period), SD = standard deviation.

### 3.1 Atrophy

**Fig. 1:**
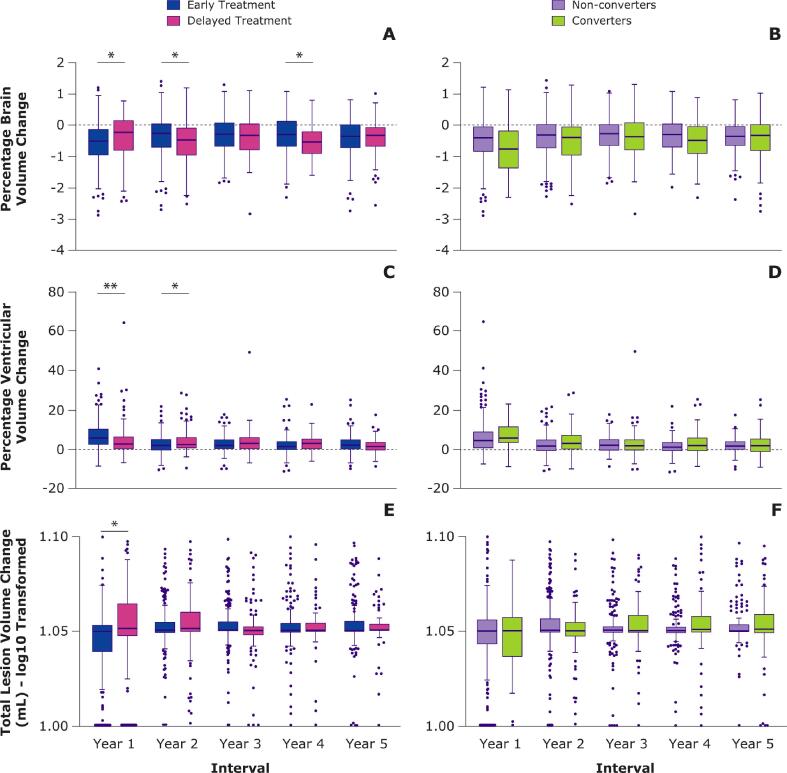
**Percentage of brain and ventricular volume changes and total lesion volume change across all years.** Boxplots depicting the percentage brain volume change, percentage ventricular volume change, and total lesion volume change (TLVC) across all years for the early and delayed treatment groups and interval-specific converters and non-converters. For visualization purposes, TLVC values were log-transformed. Within separate years, only the difference between the treatment groups was tested. Outliers beyond the y-axis range are shown on the x-axis (panel E and F). *p < 0.05. **p < 0.001.

Fig. 1, panels A and C, and Supplementary Table 3, provide the yearly PBVC and PVVC for the different treatment groups. When looking separately within each yearly interval, global atrophy was significantly faster in the first year for early versus delayed treatment (PBVC: B = − 0.198, SE = 0.069, p = 0.004), indicative of resolving edema and pseudo-atrophy. In the second and fourth year, global atrophy rate was significantly slower in the early versus delayed treatment group (PBVC, year 2: B = 0.159, SE = 0.069, p = 0.021; PBVC, year 4: B = 0.176, SE = 0.076, p = 0.021). For central atrophy, again significantly faster atrophy in the early versus delayed treatment group was found in year 1 (PVVC: B = 2.538, SE = 0.616, p < 0.001) and the opposite in year 2 (PVVC: B = − 1.560, SE = 0.614, p = 0.011). Full results are provided in Supplementary Table 4. Assessing the total effect of treatment group in the linear mixed model across the whole 5-year study period, yearly atrophy rates (PBVC and PVVC) did not differ significantly between patients in the early and delayed treatment groups. Similarly, voxel-wise analyses showed overlapping anatomical patterns of faster (pseudo) atrophy in the early treatment group in year 1 corresponding to ventricular widening and temporal lobe shrinking (V = 4165), and of faster atrophy in the delayed treatment group in year 2 corresponding to frontal lobe shrinking (V = 807). Fig. 1, panels B and D, and Supplementary Table 3, provide the yearly PBVC and PVVC for the interval-specific converters/non-converters to CDMS. Assessing the total effect of conversion in the linear mixed model across the whole 5-year study period, compared to non-converters, patients who converted to CDMS showed faster yearly global (PBVC: B = − 0.112, SE = 0.035, p = 0.001) but not central atrophy rates. In voxel-wise analyses, patients who converted during an interval showed faster atrophy compared with non-converters in years 1 (V = 1720), 2 (V = 93), and 4 (V = 919).

### 3.2 White matter lesion volume changes

In Fig. 1, panel E, and Supplementary Table 3, the yearly TLVC is provided for the early and delayed treatment groups. When looking separately within each interval, the early treatment group showed a lower TLVC compared with the delayed treatment group only in year 1 (B = − 0.318, SE = 0.125, p = 0.011). Full results are provided in Supplementary Table 4. Assessing the total effect of treatment group in the linear mixed model across the whole 5-year study period showed that TLVC did not differ significantly between the early and delayed treatment groups. In voxel-wise analyses, early treatment patients showed lower activity of enlarging lesions compared with the delayed treatment group in year 1 in the forceps minor and anterior thalamic radiation (V = 49), and higher activity of shrinking lesions in year 5 in the posterior corona radiata (V = 98).

Yearly TLVC is provided for converters/non-converters using the interval-specific CDMS status in Fig. 1, panel F, and Supplementary Table 3. Assessing the total effect of conversion in the linear mixed model across the whole 5-year study period, converters showed a significantly higher TLVC compared with non-converters (B = 0.217, SE = 0.062, p < 0.001). In year 5, converters showed higher activity of enlarging lesions compared with non-converters in the superior and posterior corona radiata, and the forceps major (V = 358).

### 3.3 Relationship between white matter lesion volume changes and subsequent atrophy

**Fig. 2:**
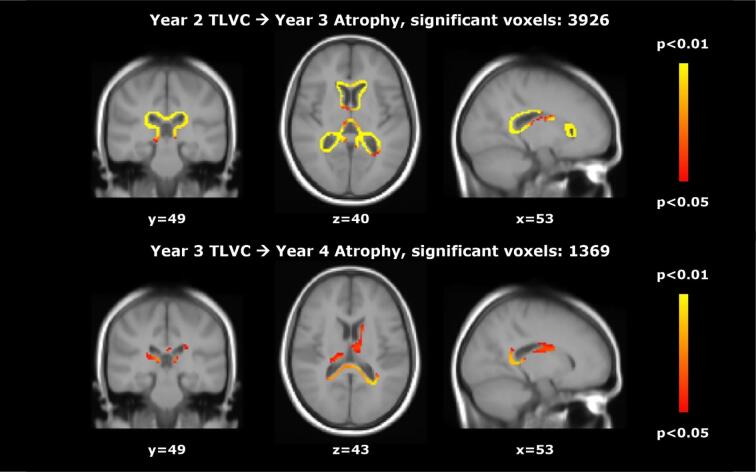
**Relationship between total lesion volume change and subsequent brain atrophy in the early treatment group.** Voxel-wise analyses in the early treatment group: significant regions where higher total lesion volume change (TLVC) in year 2 (top row) and year 3 (bottom row) was related to faster atrophy in the next year.

**Fig. 3:**
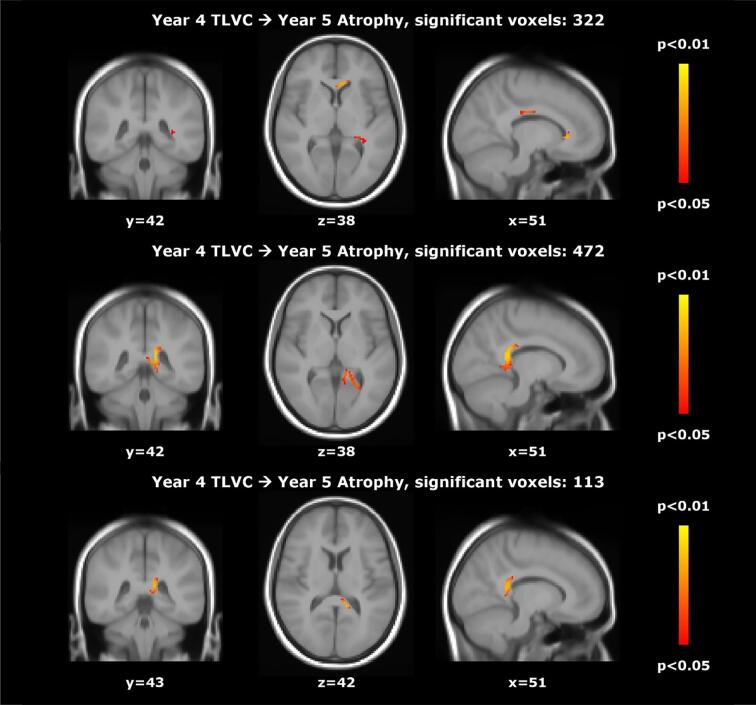
**Relationship between total lesion volume change and subsequent brain atrophy.** Voxel-wise results showing the relationship between total lesion volume change (TLVC) in year 4 and subsequent atrophy in year 5 in the early treatment group (top row) and the delayed treatment group (middle row), as well as the regions in which the relationship between TLVC and subsequent atrophy was significantly stronger in the delayed versus early treatment group (bottom row).

During stable treatment, after at least one year of treatment (so excluding the first year for the early treatment group and the third year for the delayed treatment group), we found a significant inverse relationship between TLVC and PBVC in the next year (B = − 0.113, SE = 0.022, p < 0.001), consistent with a higher lesion volume change being related to faster atrophy in the next year. This relationship did not differ significantly between the treatment groups and also not between patients who did and did not convert to CDMS across the whole study period. We found a similar significant relationship for central atrophy: TLVC was positively related to PVVC in the next year (B = 1.156, SE = 0.164, p < 0.001). This relationship did not differ significantly between converters and non-converters, but treatment seemed to moderate the effect (TLVC*treatment: B = 0.972, SE = 0.421, p = 0.021). The model showed that TLVC was only significantly related to PVVC in the next year in the early treatment group (B = 1.348, SE = 0.181, p < 0.001). Voxel-wise analyses showed that, in early treatment patients, higher TLVC in years 2 (V = 3926) and 3 (V = 1369) were related to faster atrophy in the next year, as shown in Fig. 2. In year 4, higher TLVC was related to faster atrophy in the next year in the early (V = 322) and delayed treatment (V = 472) groups. A separate analysis that included an interaction term indicated that this relationship was stronger in the delayed treatment group (V = 113), as shown in Fig. 3. The (spatio-temporal) relationship between TLVC and atrophy in the next year did not differ between CDMS converters and non-converters.

In the untreated period of patients who received delayed treatment, there was no significant relationship between TLVC and PBVC or PVVC in the next year. This relationship also did not differ significantly between the REFLEX and REFLEXION period. In voxel-wise analyses, the relationship was also not present in the REFLEX period; but in the REFLEXION period, higher TLVC in year 4 was related to faster atrophy in the next year (V = 472).

### 3.4 Relationship between atrophy and subsequent white matter lesion volume changes

During stable treatment, both global and central atrophy were associated with TLVC in the next year (PBVC: B = − 0.136, SE = 0.062, p = 0.028; PVVC: B = 0.028, SE = 0.008, p < 0.001). This relationship did not differ between patients in the two treatment groups and between CDMS converters and non-converters across the whole study period.

In patients who received delayed treatment, the relationship between PBVC and TLVC in the next year was significantly different between the REFLEX and REFLEXION study periods (TLVC*period: B = 0.411, SE = 0.207, p = 0.050). The model showed that the relationship was not significant within the periods but that the direction of the relationship was different: negative while receiving placebo in REFLEX (B = − 0.204, SE = 0.143, p = 0.158) and positive while receiving active treatment in REFLEXION (B = 0.207, SE = 0.168, p = 0.219). For PVVC, the relationship was significant in the REFLEX period (B = 0.030, SE = 0.012, p = 0.013), however, the two periods did not differ significantly.

## 4 Discussion

This study found that in patients with CIS and early MS, during stable treatment (at least one year) with sc IFN β-1a, higher WM lesion volume changes were related to faster atrophy in the next year. Interestingly, the reverse relationship was also observed: faster global and central atrophy were related to higher lesion volume changes in the next year. In the short untreated period for patients with delayed treatment, the relationship between lesion volume changes and atrophy in the next year was not significant, while faster central atrophy but not global atrophy was related to higher lesion volume changes in the next year.

Patients who received early treatment showed, during year 1, faster brain atrophy (in line with expected pseudo-atrophy) and lesion volume decrease (in line with expected resolving edema), compared with patients who received delayed treatment. In years 2 and 4, conversely, patients who received early treatment showed slower atrophy than those receiving delayed treatment. Voxel-wise analyses, similarly, showed overlapping anatomical patterns of faster ventricular widening and temporal lobe atrophy in year 1 in the early treatment group, and of faster atrophy in the frontal lobe in year 2 in the delayed treatment group.

Previous studies found that lesion measures were associated with subsequent atrophy (Dalton et al., 2002, Paolillo et al., 2004, Varosanec et al., 2015). Our results largely confirmed this: higher WM lesion volume changes were related to faster atrophy in the next year in patients with CIS and early MS. The association was not the same for all investigated subsets of the study data. We first looked at the stable treatment period, in which we only included the data points where patients had received at least 1 year of therapy. This selection was made to prevent the confounding effects of resolving edema and pseudo-atrophy during the first year of treatment with sc IFN β-1a. Anti-inflammatory medication is known to induce an initial reduction in brain volume during the first 6 months to 1 year, which is not associated with a loss of cell structures but rather fluid shifts (De Stefano et al., 2014, Zivadinov et al., 2008). The relationship between WM changes and subsequent atrophy was found in this stable treatment period. However, we did not observe this relationship in untreated patients, i.e., when we focused on the placebo period of the delayed treatment group in the first 2 years of the REFLEX study.

Our findings do not imply that treatment triggers a relationship between WM changes and subsequent atrophy. It might be expected that this relationship would be more apparent in the delayed treatment group, while these patients were receiving placebo, since the inflammation is not (yet) suppressed. However, this (exploratory) analysis only included one data point (TLVC at year 1 and PBVC or PVVC at year 2), had a relatively small sample size, and this group also has the potential for bias, because of the presumably non-random removal of patients who converted to CDMS and who were then treated with open-label sc IFN β-1a; all of these factors may have prevented the detection of a relationship in the placebo period. Since patients were recruited just after their first attack, during the first two years (the REFLEX period), placebo recipients may have exhibited shifts in fluid and changes in the volume of inflammatory cells that might obscure any relationship between true lesion accrual and true atrophy during this period. Moreover, the relationship did not differ between the REFLEX and REFLEXION period for the delayed treatment patients, meaning that there was no difference between the untreated and treated period for such patients. Therefore, treatment does not cause the association between WM changes and subsequent atrophy to appear.

For central atrophy, the relationship between WM changes and subsequent atrophy seemed to be moderated by treatment with sc IFN β-1a, since the model showed that it was only present in the early treatment group. However, this additional interaction analysis is limited by the fact that the delayed treatment patients only had one data point included (because they started treatment in year 3, unless they converted before) and the early treatment group was overrepresented with three data points for each patient (see Supplementary Table 2). Therefore, this result should be interpreted with caution.

The few studies that have investigated the relationship between WM lesions and brain atrophy in patients with CIS and early MS have focused on the hypothesis that inflammation precedes neurodegeneration. Interestingly, we also found that faster atrophy was associated with higher lesion volume changes in the next year. We found this for both global and central atrophy in the stable treatment period but only for central atrophy in the untreated period. To the best of our knowledge, this has not been investigated in a similar longitudinal manner before. Whether there is a pathological explanation for this association remains to be elucidated. One could hypothesize that if WM outside focal lesions is damaged, suffering from myelin and axonal degradation, it may be more susceptible to the formation of new focal lesions or to the expansion of pre-existing lesions. Such damage to non-lesional WM could result either from any primary degenerative process or from secondary effects of focal lesions through Wallerian degeneration, which would ultimately also lead to neuronal cell death (Filippi et al., 2019), which in turn could trigger inflammatory autoimmune responses. These changes would be indirectly observable as a loss of brain volume, thus potentially explaining the association with subsequent lesion volume changes. Alternatively, both lesion accrual and atrophy could be largely independent processes that both occur at increased rates in some patients or during periods with more severe disease. Thus, the relationships that we have observed could also be largely patient bound phenomena: if some patients steadily acquire more atrophy and lesions, while other patients steadily acquire less atrophy and lesions, this leads to correlations between atrophy and lesion changes in consecutive intervals at the group level, without there actually being any causal relationship. Future studies should investigate both hypothesized causal relationships between lesions and atrophy, as well as the possibility of correlated but not causally linked changes. Such studies should employ imaging techniques that are able to zoom in on the local microstructure and tissue properties, such as diffusion tensor imaging. Furthermore, the relationship between concurrent changes in lesions and atrophy should be investigated.

As a result of these considerations, it is interesting to observe that both the association between lesion volume changes and subsequent atrophy, and that between atrophy and subsequent lesion volume changes, were observed during stable treatment. This suggests that anti-inflammatory medication does not seem to stop the underlying pathology completely, which is not surprising, considering that interferons are first line treatment.

Treatment with sc IFN β-1a only had a significant effect on atrophy and lesion volume changes during certain periods of the study. The results in the first year were indicative of resolving edema and pseudo-atrophy, which is to be expected based on what is known about the effects of anti-inflammatory treatment during the first year (De Stefano et al., 2014, Zivadinov et al., 2008). Interestingly, we did not see this effect in the delayed treatment group in the first year of treatment (i.e., year 3 of the study). This might be because this group is smaller, but we did find a significant difference in the fourth year of the study, which could be speculated to reflect a delayed pseudo-atrophy effect in such patients. It would have been easier to explain if this effect occurred in the third year of the study, since the delayed treatment patients started treatment at that time (unless they converted to CDMS beforehand). In the fourth year, these patients had already received at least 1 year of treatment and it seems counterintuitive that the atrophy rate had not become more similar between the early and delayed treatment groups by then. This potentially has an important clinical consequence, by highlighting a different response to early and delayed treatment in terms of brain atrophy and lesion accrual.

Patients who converted to CDMS showed faster global atrophy and higher lesion volume changes across the whole study period compared to non-converters, and since these patients have a worse disease progression this seems to be expected. However, because they received treatment with sc IFN β-1a three times a week upon conversion to CDMS, this result is somewhat difficult to interpret.

### Limitations:

Since the study design is complicated because treatment (dosing) and conversion status are intertwined, it was not possible to correct for all potential confounders. We tried to account for this in the linear mixed models, and for this reason we analyzed specific subsets of the study data. For example, to look at a pure untreated placebo group we excluded the converters in the first and second year of the study, but this also introduced a potential selection bias of including only the cases with a more benign disease progression.

Due to the explorative nature of the post-hoc analyses, a correction for multiple testing was not performed. Therefore, the results from the current study need to be confirmed in other datasets in future studies and should be interpreted with caution.

Ideally, we would have looked at the relationship between WM lesions and gray matter (GM) atrophy. However, due to the quality of the 2D scans, with limited contrast in the images, which makes it difficult to detect the WM-GM border, this was not possible. For this reason, we focused on global and central atrophy measures, and we cannot make statements about the relationship between WM and GM pathology in MS. Future studies using 3D imaging with adequate WM-GM contrast could address these issues, including the relationship between specific cortical lobes and WM tracts in the brain. Moreover, besides GM changes, diffuse WM changes may also contribute to the evolution of brain atrophy (Ceccarelli et al., 2007, Kalkers et al., 2002). In the present dataset however, we were only able to investigate the effect of focal lesions.

A strength of this study was regular, tightly controlled MRI scans over a fairly long period, and a large sample that enabled us to investigate the relationship between atrophy and WM changes in patients with CIS early in the disease process. Ideally these patients would have been followed-up for an even longer duration. Therefore, future studies should aim to increase the follow-up period to better elucidate the relationship between the two pathological processes, and make use of the most recent advances in imaging such as 3D-FLAIR and 3D-T1 images to be able to look in more detail at GM and WM; for example, by measuring cortical thickness. These studies should also focus on WM damage potentially being secondary to neurodegeneration.

## 5 Conclusions

Higher lesion volume changes were related to faster atrophy in the next year in patients with CIS and early MS, and vice versa, faster atrophy was related to subsequent higher lesion volume changes. The question remains whether these processes are causally related or whether they are merely two pathological processes that occur simultaneously in such patients. This needs to be investigated further in future studies.

## 6 Data availability statement

Any requests for data by qualified scientific and medical researchers for legitimate research purposes will be subject to Merck Healthcare KGaA’s Data Sharing Policy. All requests should be submitted in writing to Merck Healthcare KGaA’s data sharing portal https://www.merckgroup.com/en/research/our-approach-to-research-and-development/healthcare/clinical-trials/commitment-responsible-data-sharing.html. When Merck Healthcare KGaA has a co-research, co-development, or co-marketing or co-promotion agreement, or when the product has been out-licensed, the responsibility for disclosure might be dependent on the agreement between parties. Under these circumstances, Merck Healthcare KGaA will endeavor to gain agreement to share data in response to requests.

## Funding

The REFLEXION study was supported by Merck (CrossRef Funder ID: 10.13039/100009945).

## CRediT authorship contribution statement

**Rozemarijn M. Mattiesing:** Conceptualization, Formal analysis, Investigation, Methodology, Visualization, Writing – original draft, Writing – review & editing. **Giordano Gentile:** Conceptualization, Formal analysis, Investigation, Methodology, Visualization, Writing – original draft, Writing – review & editing. **Iman Brouwer:** Investigation, Methodology, Software, Writing – review & editing. **Ronald A. van Schijndel:** Investigation, Methodology, Software, Writing – review & editing. **Bernard M.J. Uitdehaag:** Writing – review & editing. **Jos W.R. Twisk:** Methodology, Writing – review & editing. **Ludwig Kappos:** Writing – review & editing. **Mark S. Freedman:** Writing – review & editing. **Giancarlo Comi:** Writing – review & editing. **Dominic Jack:** Writing – review & editing. **Nicola De Stefano:** Conceptualization, Funding acquisition, Investigation, Supervision, Writing – review & editing. **Frederik Barkhof:** Conceptualization, Funding acquisition, Investigation, Supervision, Writing – review & editing. **Marco Battaglini:** Conceptualization, Funding acquisition, Investigation, Methodology, Supervision, Writing – original draft, Writing – review & editing. **Hugo Vrenken:** Conceptualization, Funding acquisition, Investigation, Methodology, Supervision, Writing – original draft, Writing – review & editing.

## Declaration of Competing Interest

**RMM** has received research support from Merck. **IB** has received research support from Merck, Novartis, Teva, and the Dutch MS Research Foundation. **BMJU** reports research support and/or consultancy fees from Biogen Idec, Genzyme, Merck Serono, Novartis, Roche, Teva, and Immunic Therapeutics. **LK's** institution (University Hospital Basel) has received the following exclusively for research support: Steering committee, advisory board, and consultancy fees (Actelion [Janssen/J&J], Bayer, Biogen, BMS, Genzyme, Janssen, Merck, Novartis, Roche, Sanofi, Santhera, and TG Therapeutics); speaker fees (Bayer, Biogen, Merck, Novartis, Roche, and Sanofi); support of educational activities (Allergan, Bayer, Biogen, CSL Behring, Desitin, Genzyme, Merck, Novartis, Roche, Pfizer, Sanofi, Shire, and Teva); license fees for Neurostatus products; and grants (Bayer, Biogen, European Union, InnoSwiss, Merck, Novartis, Roche, Swiss MS Society, and Swiss National Research Foundation). **MSF** has received honoraria or consultation fees from Alexion, Atara Biotherapeutics, Bayer, BeiGene, BMS (Celgene), EMD Inc., Canada (an affiliate of Merck KGaA, Darmstadt, Germany), Hoffmann La-Roche, Janssen (J&J), Merck KGaA (Darmstadt, Germany), Novartis, Pendopharm, and Sanofi-Genzyme; has been a member of a company advisory board, board of directors, or other similar group for Alexion, Atara Biotherapeutics, Bayer, BeiGene, BMS (Celgene), Clene Nanomedicine, Hoffmann La-Roche, Janssen (J&J), McKesson, Merck KGaA (Darmstadt, Germany), Novartis, and Sanofi-Genzyme; has participated in a company sponsored speaker’s bureau for EMD Serono Research & Development Institute, Inc., Billerica, MA, USA (an affiliate of Merck KGaA) and Sanofi-Genzyme; and has been in receipt of research or educational grants from Sanofi-Genzyme. **GC** has received consulting fees from Bayer, Biogen, Merck, Novartis, Receptos, Roche/Genentech, Sanofi-Aventis, and Teva Pharmaceutical Industries Ltd; lecture fees from Bayer, Biogen, Merck KGaA, Novartis, Sanofi-Aventis, Serono Symposia International Foundation, and Teva Pharmaceutical Industries Ltd; and trial grant support from Bayer, Biogen, Merck, Novartis, Receptos, Roche/Genentech, Sanofi-Aventis, and Teva Pharmaceutical Industries Ltd. **DJ** is an employee of Merck Serono Ltd, Feltham, UK (an affiliate of Merck KGaA). **NDeS** is a consultant for Biogen, Merck, Novartis, Roche, Sanofi-Genzyme, and Teva; has grants or grants pending from FISM and Novartis, is on the speakers’ bureaus of Biogen, Merck, Novartis, Roche, Sanofi-Genzyme, and Teva; and has received travel funds from Merck, Novartis, Roche, Sanofi-Genzyme, and Teva. **FB** is supported by the NIHR Biomedical Research Center at UCLH and is a consultant to Biogen, Combinostics, IXICO, Merck, and Roche. **HV** has received research support from Merck, Novartis, Pfizer, and Teva, consulting fees from Merck, and speaker honoraria from Novartis; all funds were paid to his institution. **GG, RAvS, JWRT,** and **MB** report no disclosures.
