# Supplementary material for: The spatio-temporal relationship between white matter lesion volume changes and brain atrophy in clinically isolated syndrome and early multiple sclerosis
Source: Neuroimage Clin. 2022 Oct 3;36:103220. doi: 10.1016/j.nicl.2022.103220 (PMC9668617; doi:10.1016/j.nicl.2022.103220)
Supplement: Supplementary material 1 [file mmc1.pdf]

Supplementary material

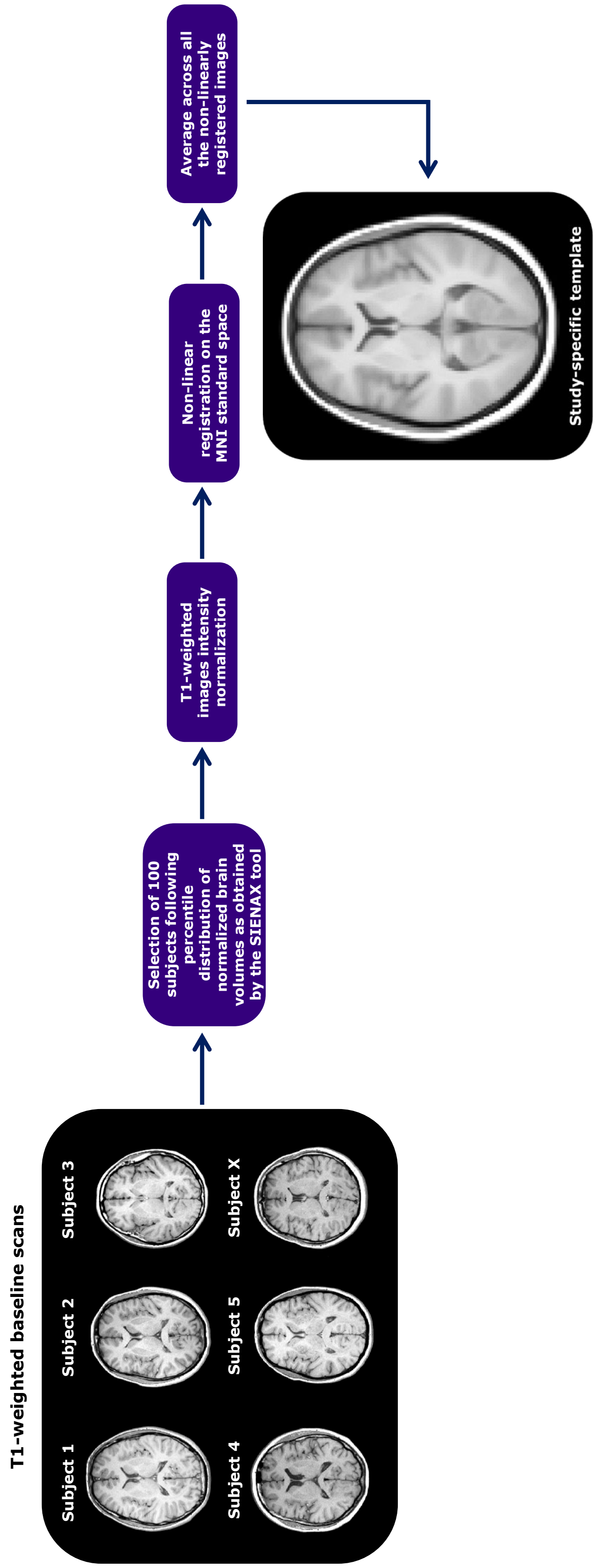

Supplementary Figure 1. Schematized depiction of the creation of the study-specific template as explained in section 2.5 of the main manuscript.

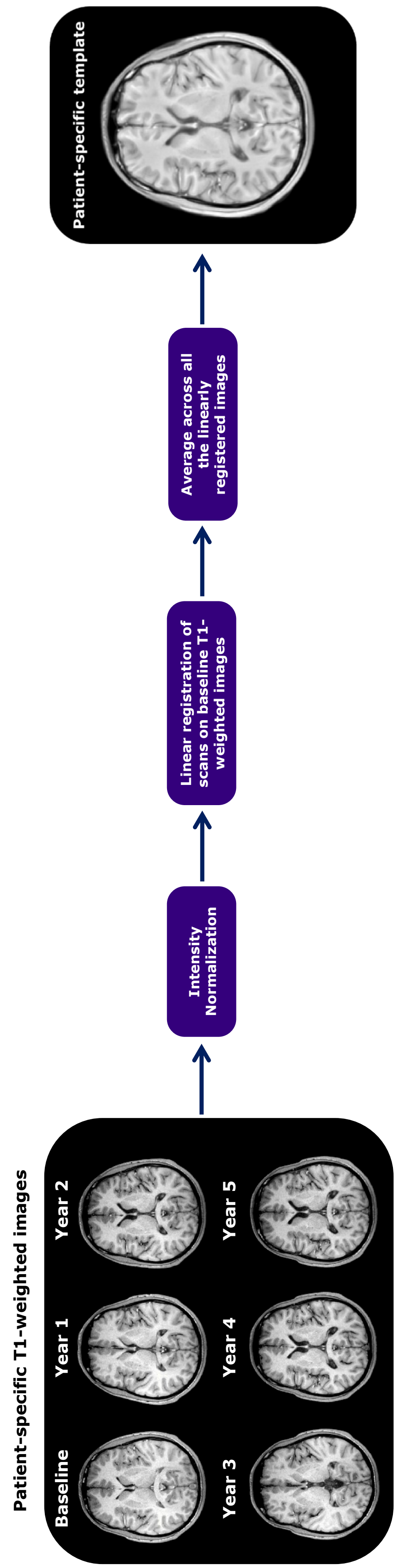

Supplementary Figure 2. Schematized depiction of the creation of a patient-specific template as explained in section 2.5 of the main manuscript.

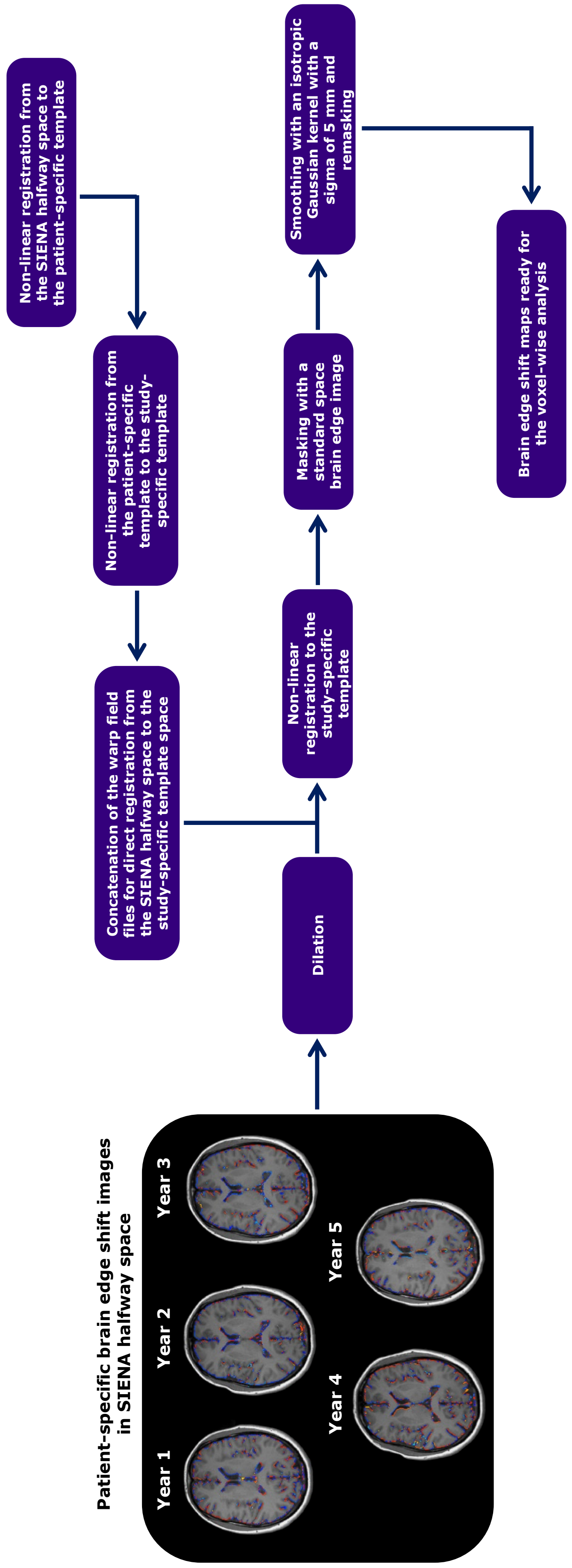

Supplementary Figure 3. Schematized depiction of the atrophy voxel-wise analysis pipeline as explained in section 2.5 of the main manuscript.

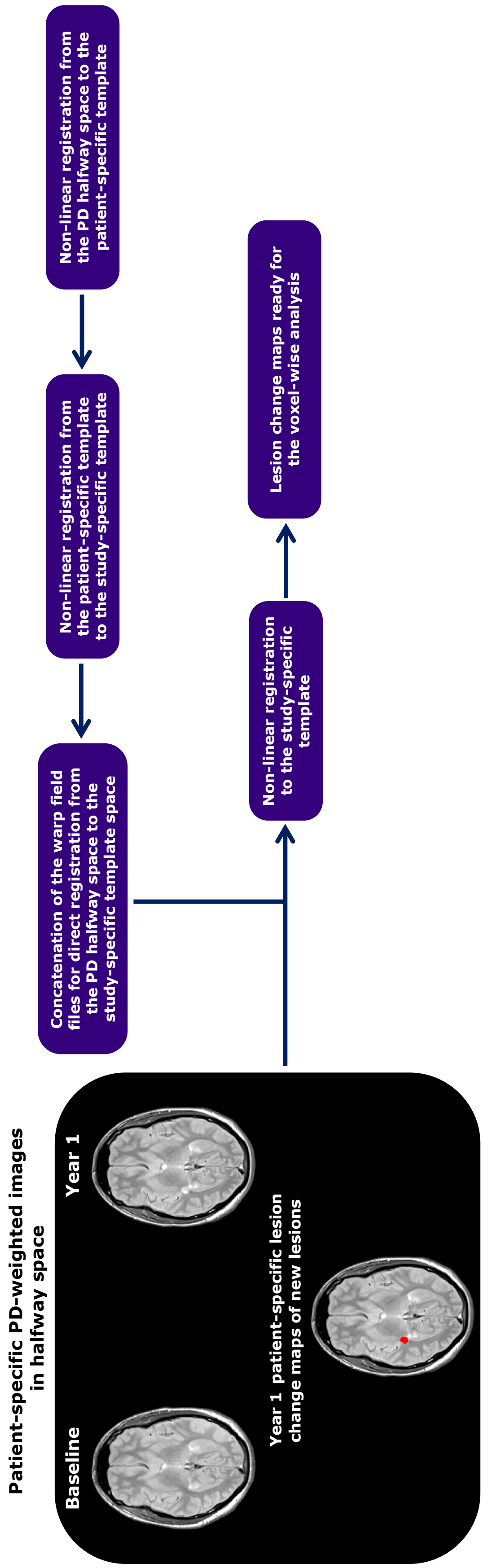

Supplementary Figure 4. Schematized depiction of the lesion activity voxel-wise analysis pipeline as explained in section 2.5 of the main manuscript.
For the sake of simplicity only new lesion activity is shown. PD = proton-density.

Supplementary Table 1. Linear mixed models with (repeated) yearly measurements.

Model	Period	Time-lag model?	Dependent variable	Independent variables	Interaction term
1	Whole study	No	PBVC	Sex, Age, Interval-specific CDMS status, Treatment	N/A
2	Whole study	No	PVVC	Sex, Age, Interval-specific CDMS status, Treatment	N/A
3	Whole study	No	TLVC	Sex, Age, Interval-specific CDMS status, Treatment	N/A
4	Whole study	No	PBVC	Sex, Age, Interval-specific CDMS status, Treatment, Interval	Treatment*Interval
5	Whole study	No	PVVC	Sex, Age, Interval-specific CDMS status, Treatment, Interval	Treatment*Interval
6	Whole study	No	TLVC	Sex, Age, Interval-specific CDMS status, Treatment, Interval	Treatment*Interval
7	Stable treatment	Yes	PBVC	TLVC, Sex, Age, Overall CDMS status, Treatment	N/A
8	Stable treatment	Yes	PVVC	TLVC, Sex, Age, Overall CDMS status, Treatment	N/A
9	Stable treatment	Yes	PBVC	TLVC, Sex, Age, Overall CDMS status, Treatment	TLVC*Treatment
10	Stable treatment	Yes	PVVC	TLVC, Sex, Age, Overall CDMS status, Treatment	TLVC*Treatment
11	Stable treatment	Yes	PBVC	TLVC, Sex, Age, Overall CDMS status, Treatment	TLVC*Overall CDMS status
12	Stable treatment	Yes	PVVC	TLVC, Sex, Age, Overall CDMS status, Treatment	TLVC*Overall CDMS status
13	DT untreated	Yes	PBVC	TLVC, Sex, Age, Overall CDMS status#	N/A
14	DT untreated	Yes	PVVC	TLVC, Sex, Age, Overall CDMS status#	N/A
15	DT untreated vs treated	Yes	PBVC	TLVC, Sex, Age, Overall CDMS status, Period	TLVC*Period
16	DT untreated vs treated	Yes	PVVC	TLVC, Sex, Age, Overall CDMS status, Period	TLVC*Period
17	Stable treatment	Yes	TLVC	PBVC, Sex, Age, Overall CDMS status, Treatment	N/A
18	Stable treatment	Yes	TLVC	PVVC, Sex, Age, Overall CDMS status, Treatment	N/A
19	Stable treatment	Yes	TLVC	PBVC, Sex, Age, Overall CDMS status, Treatment	PBVC*Treatment
20	Stable treatment	Yes	TLVC	PVVC, Sex, Age, Overall CDMS status, Treatment	PVVC*Treatment
21	Stable treatment	Yes	TLVC	PBVC, Sex, Age, Overall CDMS status, Treatment	PBVC*Overall CDMS status
22	Stable treatment	Yes	TLVC	PVVC, Sex, Age, Overall CDMS status, Treatment	PVVC*Overall CDMS status

23	DT untreated	Yes	TLVC	PBVC, Sex, Age, Overall CDMS status#	N/A
24	DT untreated	Yes	TLVC	PVVC, Sex, Age, Overall CDMS status#	N/A
25	DT untreated vs treated	Yes	TLVC	PBVC, Sex, Age, Overall CDMS status, Period	PBVC*Period
26	DT untreated vs treated	Yes	TLVC	PVVC, Sex, Age, Overall CDMS status, Period	PVVC*Period

In each model a three level structure was incorporated where observations were clustered within patients and patients were clustered within study sites, except for # where only one measurement for each patient was included, so only patients were clustered within study sites. CDMS = clinically definite multiple sclerosis, DT = delayed treatment, N/A = not applicable, PBVC = percentage brain volume change, PVVC = percentage ventricular volume change, TLVC = total lesion volume change. The different periods and CDMS status categories are explained in the main text.

Supplementary Table 2. Time-lag model and the selection of data points for the stable treatment period, and for the untreated and treated periods of the delayed treatment group.

**Interval for independent variable (TLVC)**	**Interval for dependent variable (PBVC or PVVC)**	**Use of data points**			
		**Stable treatment analysis**		**Analysis of untreated, and untreated vs treated period in the delayed treatment group**	
		**Early treatment**	**Delayed treatment**	**Untreated**	**Untreated vs treated**
Year 1	Year 2			X	X
Year 2	Year 3	X			
Year 3	Year 4	X			
Year 4	Year 5	X	X		X

This table shows the shift in time in the dependent variable, allowing to analyze the relationship between total lesion volume change (TLVC) in a study year and global (PBVC) or central (PVVC) atrophy measures in the next year, and vice versa with PBVC or PVVC as the independent variable and TLVC as the dependent variable.

Supplementary Table 3. Longitudinal atrophy and lesion volume change measures across treatment groups and interval-specific converters to clinically definite multiple sclerosis and non-converters, in each year of the study.

Measure	Group	Year 1	Year 2	Year 3	Year 4	Year 5
PBVC (%/y)	ET	-0.541±0.722	-0.353±0.603	-0.322±0.547	-0.311±0.584	-0.412±0.597
	DT	-0.362±0.675	-0.536±0.750	-0.371±0.591	-0.508±0.533	-0.416±0.573
	Nonc	-0.443±0.685	-0.375±0.620	-0.324±0.522	-0.316±0.525	-0.382±0.518
	Conv	-0.752±0.828	-0.521±0.754	-0.366±0.633	-0.480±0.640	-0.464±0.687
PVVC (%/y)	ET	6.868±7.095	2.373±4.582	2.518±4.195	1.968±4.725	2.602±4.354
	DT	4.413±8.479	4.022±5.965	3.604±6.394	2.901±4.431	1.850±3.933
	Nonc	5.870±7.741	2.412±4.620	2.843±4.186	1.713±3.935	2.098±3.435
	Conv	7.283±7.045	4.319±6.149	2.941±6.414	3.254±5.540	2.816±5.270
TLVC (mL/y)	ET	-0.339±1.568	0.207±0.983	0.214±0.837	0.150±1.002	0.185±0.874
	DT	-0.006±1.945	0.218±0.838	0.065±0.960	0.222±1.078	0.041±0.697
	Nonc	-0.254±1.731	0.217±0.927	0.097±0.664	0.077±0.643	0.073±0.542
	Conv	-0.057±1.556	0.196±0.961	0.296±1.205	0.345±1.469	0.250±1.135

Values are mean ± standard deviation. Conv = converters to clinically definite multiple sclerosis (CDMS), DT = delayed treatment, ET = early treatment, Nonc = non-converters to CDMS, PBVC = percentage brain volume change, PVVC = percentage ventricular volume change, TLVC = total lesion volume change.

Supplementary Table 4. Statistical analysis of the differences in atrophy and lesion change measures between the early and delayed treatment groups in each year of the study.

Interval	PBVC	PVVC	TLVC
Year 1	B=-0.198, SE=0.069, p=0.004	B=2.538, SE=0.616, p<0.001	B=-0.318, SE=0.125, p=0.011
Year 2	B=0.159, SE=0.069, p=0.021	B=-1.560, SE=0.614, p=0.011	B=0.014, SE=0.124, p=0.912
Year 3	B=0.024, SE=0.074, p=0.751	B=-0.841, SE=0.658, p=0.201	B=0.171, SE=0.128, p=0.184
Year 4	B=0.176, SE=0.076, p=0.021	B=-0.778, SE=0.675, p=0.249	B=-0.056, SE=0.135, p=0.676
Year 5	B=-0.005, SE=0.079, p=0.945	B=0.799, SE=0.702, p=0.255	B=0.151, SE=0.143, p=0.292

PBVC = percentage brain volume change, PVVC = percentage ventricular volume change, TLVC = total lesion volume change.
